# Harnessing the Neural Stem Cell Secretome for Regenerative Neuroimmunology

**DOI:** 10.3389/fncel.2020.590960

**Published:** 2020-11-05

**Authors:** Cory M. Willis, Alexandra M. Nicaise, Regan Hamel, Vasiliki Pappa, Luca Peruzzotti-Jametti, Stefano Pluchino

**Affiliations:** Department of Clinical Neurosciences and NIHR Biomedical Research Centre, University of Cambridge, Cambridge, United Kingdom

**Keywords:** stem cell secretome, neural stem cells, immune modulation, CNS injury, extracellular vesicles, regenerative neuroimmunology, COVID-19 and cytokine storm syndrome

## Abstract

Increasing evidence foresees the *secretome* of neural stem cells (NSCs) to confer superimposable beneficial properties as exogenous NSC transplants in experimental treatments of traumas and diseases of the central nervous system (CNS). Naturally produced *secretome* biologics include membrane-free signaling molecules and extracellular membrane vesicles (EVs) capable of regulating broad functional responses. The development of high-throughput screening pipelines for the identification and validation of NSC *secretome* targets is still in early development. Encouraging results from pre-clinical animal models of disease have highlighted *secretome*-based (acellular) therapeutics as providing significant improvements in biochemical and behavioral measurements. Most of these responses are being hypothesized to be the result of modulating and promoting the restoration of key inflammatory and regenerative programs in the CNS. Here, we will review the most recent findings regarding the identification of NSC-secreted factors capable of modulating the immune response to promote the regeneration of the CNS in animal models of CNS trauma and inflammatory disease and discuss the increased interest to refine the pro-regenerative features of the NSC *secretome* into a clinically available therapy in the emerging field of Regenerative Neuroimmunology.

## Introduction

The development of non-hematopoietic stem cell-based therapies for the treatment of diseases of the central nervous system (CNS) has seen major recent advances, with many of these therapies undergoing early-phase clinical testing of feasibility and safety for the treatment of a wide range of neurodegenerative diseases (Trounson and McDonald, 2015; Pluchino et al., 2020). Advances within the field of neural stem cell (NSC) biology, and building from early, positive outcomes of transplantation studies in experimental animal disease models, has firmly placed NSCs at the forefront for the development of clinically applicable exogenous stem cell therapies (L’Episcopo et al., 2018; Sullivan et al., 2020; Willis et al., 2020).

Evidence gathered thus far has identified a dual role for transplanted NSCs in fostering regeneration within the damaged and diseased CNS. On the one hand, transplanted NSCs can generate graft-derived neurons and glial cells (Martino and Pluchino, 2006; Martino et al., 2011; Boese et al., 2018), on the other hand, the NSC *secretome* provides a vast array of signaling molecules, including growth factors, cytokines, chemokines, metabolites, and bioactive lipids (Drago et al., 2013; Hicks et al., 2013; Shoemaker and Kornblum, 2016) that are known to possess the capability to orchestrate multiple interactions with the surrounding microenvironment, in particular promoting changes in inflammation states (Peruzzotti-Jametti et al., 2018). Thus, developing novel, high-throughput strategies to screen NSC factors and identify targets with pro-regenerative properties is a growing area of active investigation.

The biologics of the NSC *secretome* can be broadly divided into membrane-free and membrane-enclosed secreted candidate factors. The former denotes bioactive molecules secreted through direct translocation across the plasma membrane, while the latter indicates the packaging of factors into secretory membrane vesicles consisting of liquid or cytoplasm enclosed by a lipid bilayer. Here, vesicles can be generated by direct “*shedding*” from the plasma membrane or through a complex endosome-derived process of vesicle trafficking and secretion (Abels and Breakefield, 2016). Once released, intact vesicles are known as *extracellular vesicles* (EVs). The field of EV biology has exploded within the last decade, spurring an intense interest in the multi-functional aspects of these membranous vesicles as mediators of cell-to-cell communication.

Several roles have been ascribed to NSC-EVs in influencing physiological and pathological conditions through the transfer of micro RNAs (miRNAs; Morton et al., 2018), the transfer of cytokine-receptor complexes to mediate immune signaling (Cossetti et al., 2014), and even as fully functional independent metabolic units (Iraci et al., 2017). Within this context, EVs are just now being recognized as a critical component of the NSC *secretome* with the potential to serve as a *bona fide* therapeutic adjuvant in not only the regeneration of damaged and disease CNS tissue (Vogel et al., 2018) but as potent modulators of the immune response (Rong et al., 2019).

Despite rapid advancements in understanding the biological heterogeneity of EVs, major technological limitations remain (Carpintero-Fernandez et al., 2017; Ramirez et al., 2018; Gandham et al., 2020). The biggest of which is their broad range of sizes, ranging from as small as 50 nm (termed “exosomes”) to as large as 1μm (termed “microvesicles”), which can exceed the limit of detection of many common optical-based analytical techniques, such as flow cytometry and fluorescence microscopy, making the rapid identification of individual EVs practically impossible (Margolis and Sadovsky, 2019). Further, a widely accepted and defined protocol for EV isolation is currently lacking, making cross-study comparisons challenging. This has led to the development of a minimum set of standards to report on EVs (Théry et al., 2018). Last, quantifying EVs is still highly contentious despite the implementation of particle tracking devices and commercially available kits for the determination of absolute EV numbers. Given the technological limitations inherent in EV biology, great strides have been made in codifying and formalizing the nomenclature of EV subtypes (Jeppesen et al., 2019; Witwer and Théry, 2019). Technological advancements in optical resolution and particle tracking will undoubtedly lead to a new and improved understanding of their function, with direct benefit to the clinical applicability of NSC-EVs.

In this review article, we will discuss the current methods for analyzing the *secretome*, including target identification and candidate validation of soluble vs. EV-associated factors. From there, we will explore how soluble and EV-associated NSC factors are capable of modulating the immune response to promote a pro-regenerative environment within the damaged and diseased CNS. Last, we will discuss the implication of these findings for clinical work and the benefits of NSCs in a clinical capacity.

## Secretome Analysis and Target Identification

Target screening and identification methodologies of NSC-secreted factors capable of modulating the immune response and promoting recovery in experimental animal models of CNS diseases include both unbiased and biased approaches.

**Figure 1:**
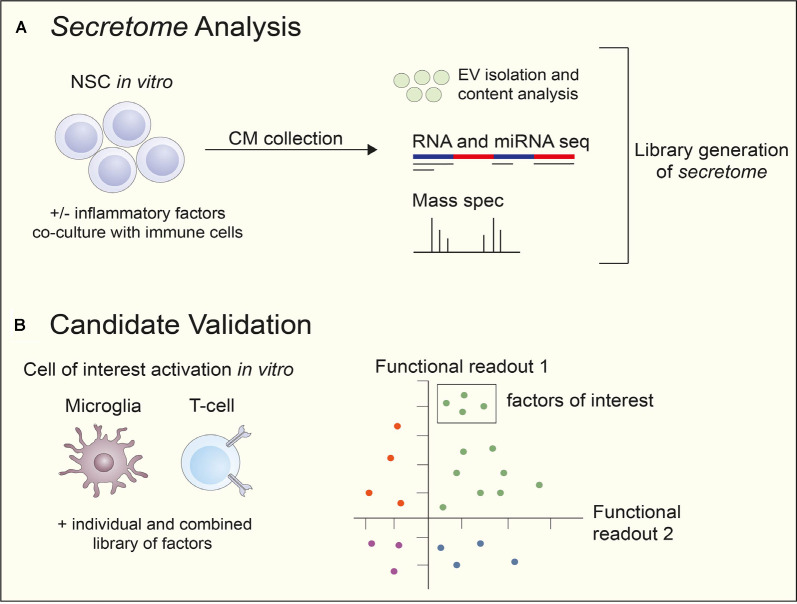
The workflow of NSC secretome analysis and candidate validation. **(A)** NSCs from various sources, rodent, human fetal and induced pluripotent stem cell (iPSC)-derived, are cultured *in vitro* with or without inflammatory factors or in a co-culture with immune cells to stimulate secretion. conditioned media (CM) are collected and unbiased analyses performed, including RNA and miRNA seq and mass spectrometry. EVs are also isolated and their contents are examined. After analysis, a library of the NSC *secretome* is generated. **(B)** After the generation of an NSC secreted factor library, the candidates are tested using cells of interest *in vitro*. Predetermined functional readouts, such as secretion of specific cytokines or upregulation of proteins are measured and secreted factors of interest narrowed down depending on cell effects. Abbreviations: CM, conditioned media; EV, extracellular vesicles; mass spec, mass spectrometry; NSC, neural stem cell; seq, sequencing.

Current unbiased approaches include quantitative proteomics on complex biological fluids, such as conditioned media (CM), and NSC-EVs *in vitro* using mass spectrometry-based technology in addition to small RNA-sequencing. Unbiased, large-scale proteomic and sequencing datasets generated through these means create repositories of the NSC *secretome*. From here, biased approaches are taken to generate screening libraries for the identification of biologically relevant targets (Figure 1A). However, one technical limitation that must be considered when performing quantitative proteomics is the lower limit of detection of analytes within the sample (Chevallet et al., 2007). For example, if the target(s) of interest is not sufficiently secreted or rapidly metabolized, it will fall below the threshold of detection of the instrument and not be identified. As such, *a priori* knowledge of the target(s) abundance is critical before performing expensive and time-consuming mass-spectrometry based profiling.

NSC-secreted factors influence and alter the phenotype of immune cells in a paracrine manner, which can establish a bidirectional feedback loop that can alter NSC behaviors (Peruzzotti-Jametti et al., 2018). Therefore, the analysis of secreted factors after these interactions is critical for understanding the underlying mechanism of action. One way this can be accomplished is through stable isotope labeling with amino acids (SILAC), which is a mass spectrometry-based technique that relies on non-radioactive isotopic labeling of cells to detect and quantify secreted factors from complex samples. Here, the pre-labeling of either NSCs or the immune cell of choice with heavy isotopes will allow for the proteomic profiling and identification of secreted factors (Hathout, 2007). In this scenario, one could imagine different co-culture conditions wherein the effects of the NSC *secretome* on target cells are profiled and proteomic datasets generated for downstream biased approaches at target identification.

*Secretome* libraries can be prepared either from soluble proteins or secreted EV components (i.e., proteins, RNA). In the latter case, proteins and RNA should be first isolated from EVs. Upon library construction, cell-based assays can be used to perform *secretome*-based screening. The most physiologically relevant cells should be used to obtain relevant results and to achieve a successful research outcome. Cells should be amenable to the assay, reliably represent the system, and express the necessary factors and signaling intermediates (Figure 1B; An and Tolliday, 2010).

In these assays, cells are usually incubated with the secretome proteins from a previously annotated *secretome library* for a few days *in vitro* to capture cell proliferation, differentiation, and *de novo* expression of specific marker proteins. Each individual factor in the *secretome library* is tested in technical replicates. Ideally, positive and negative controls should be included on each plate. These controls are used for the calculation of Z’-factor which is normally used to characterize assay performance (Ding et al., 2020). Different technologies can be used for assay readout, including fluorescence, immunoassays, mass spectrometry, qRT-PCR, flow cytometry, imaging, depending on the question to be addressed. The most common method for hit selection in screening is by considering z-score. Hits with a z-score above the threshold are selected and should be further validated. Z-score should not be confused with Z’-factor. Z’-factor is a measure of assay quality, whereas z-score, which is calculated for each factor on the plate, provides information on the strength of each factor relative to the rest of the sample distribution (Birmingham et al., 2009).

Quality control (QC) measures are crucial in screening platforms to ensure the reliability of the generated data. The utilization of QC measures proves to be beneficial since it prevents the analysis of poor quality screening data that will lead to misleading results, captures technical issues that may arise, and allows the comparison between assays (Ding et al., 2020).

Many measures have been proposed to evaluate assay quality. These can be related to plate level controls and sample level controls (Chen et al., 2016). Z’ factor is the most commonly used measure of screening assay quality indicating the “assay window”, which refers to the space between positive and negative control where screening factors should exhibit their activity. Z’-factor is calculated for each plate by considering positive and negative controls. A clear distinction between positive and negative control is an indicator of good assay quality. Thus, the selection of effective controls is crucial. A value of Z’-factor close to 1 indicates an ideal assay. An assay with a value between 0.5 > Z’ > 1 is generally considered as a good quality screening, whereas Z’ factor <0.5 means that results are questionable (Bray and Carpenter, 2004). A generalization of the Z’-factor, which is referred to as V-factor, is used as a measure of quality in a concentration-response (CR) assay (Bray and Carpenter, 2004).

Signal to background (S/B) and signal to noise (S/N) are often used as measures of assay performance. Unfortunately, these ratios do not consider assay variability risking to generate misleading results and, thus they are not appropriate in the evaluation of an assay (Zhang, 2008). Similar to Z’-factor, signal window (SW) and assay variation ratio (AVR) take assay variability into account (Zhang, 2008). Iversen et al. (2006) support that Z’ factor is more accurate and precise than SW. AVR is equivalent to Z’-factor (Zhang, 2008). The coefficient of variation (CV) performs a measure of relative dispersion of the data. Recently, strict standard mean difference (SSMD) has been proposed for assessing data quality in screening assays (Zhang, 2007, 2008). Similar to Z’-factor, SSMD characterizes the performance of the controls on an individual plate (Chen et al., 2016). However, the advantage of SSMD lies in probabilistic interpretation and statistical estimation and inference (Zhang, 2008).

Since the utility of individual QC measures is not sufficient to assess the assay quality, it is common to report multiple QC measures for a screening (Chen et al., 2016). For example, Z’-factor can be used in conjunction with CV, S/B, or S/N to give a more concise assessment of the assay variation and performance.

Once screening hits have been identified, they should be carried forward to hit validation. Thus, each hit is evaluated through multiple-point (e.g., between 7- and 10-point) CR in duplicate or triplicate in the primary cell-based assay (Wadsworth et al., 2019; Ding et al., 2020). IC/EC50 can be used as parameters for assay validation. In case human primary cells are used in assays, different donor cells need to be tested since cell variability can be expected (Ding et al., 2020). Locci et al. (2016) used cells from multiple donors to confirm activin A as a regulator of human T_FH_ cell differentiation. Upon a hit confirmation, the list of confirmed factors is annotated in silico, including literature searches, expression data, disease relevance, and human target validation (Ding et al., 2020; Figure 1).

## Modulation of Cellular Responses

### NSC-Secreted Factors and Immune Response

Dampening down persistent neuroinflammation is a key challenge in developing therapeutic approaches to many chronic neurological pathologies. In this regard, NSCs have been shown to secrete immunomodulatory factors that can alter the course of disease progression (Volpe et al., 2019). Studies from a wide range of neurological diseases have overwhelmingly concluded that NSCs have anti-inflammatory and tissue-regenerative effects on their environment (Ziv et al., 2006; Redmond et al., 2007; Chen et al., 2014; Lee et al., 2015; Cheng et al., 2017; McGinley et al., 2018; Mendes-Pinheiro et al., 2018; Peruzzotti-Jametti et al., 2018; Zalfa et al., 2019), but few studies have managed to pinpoint the exact molecules behind these effects.

A recent study from our group discovered that one of the molecules behind the immunomodulatory effects of NSCs is the eicosanoid prostaglandin E2 (PGE2; Peruzzotti-Jametti et al., 2018). We found that *in vitro* treatment of NSCs by either lipopolysaccharide (LPS) or the tricarboxylic acid cycle (Kreb’s cycle) intermediate succinate enhanced PGE2 secretion. Furthermore, PGE2 secretion was demonstrated as partially responsible for the observed phenotypic switch in microglia and macrophages from pro- to anti-inflammatory, which was accompanied by the downregulation of the pro-inflammatory cytokine *Il1b* *in vitro*. Ultimately, this work has highlighted an immunomodulatory role of NSCs through the secretion of bioactive molecules.

Cytokines are a broad category of membrane-impermeable peptides with immunomodulatory properties that are actively released by NSCs *in vitro* with implications for *in vivo* immune modulation (Liu et al., 2013). In the Theiler’s murine encephalomyelitis virus (TMEV) model of multiple sclerosis (MS), the transplantation of human fetal (h)NSCs was found to modulate the T cell-mediated immune response, as evidenced by decreased interferon-gamma (IFNγ) and tumor necrosis factor-alpha (TNFα) and increased interleukin-10 (IL-10) production from T cells isolated from draining cervical lymph nodes, and an increase in CD4^+^CD25^+^FOXP3^+^ regulatory T-cells (Chen et al., 2014). Follow-up *in vitro* co-culture studies of activated T cells and hNSCs identified reduced T-cell proliferation concomitant with increased regulatory T cell induction that was dependent on the secretion of transforming growth factor (TGF)-β1 and TGF-β2 from hNSCs as the effect was blocked by neutralizing antibodies against TGF-β1 and TGF-β2. From these *in vivo* and *in vitro* findings, the authors concluded that despite the temporary viability of the transplanted hNSCs in their viral MS model, the hNSC *secretome* provided long-lasting anti-inflammatory and regenerative effects. A follow-up study from the same group demonstrated similar results are observed from transplanted hNSCs derived from induced pluripotent stem cells (iPSCs; Plaisted et al., 2016). Furthermore, a recent study in a non-viral mouse model of MS, experimental autoimmune encephalomyelitis (EAE), demonstrated that mouse embryonic NSC-secreted TGF-β2 inhibits the differentiation of pro-inflammatory monocyte-derived dendritic cells *in vivo* and *in vitro* (De Feo et al., 2017).

While specific mechanisms relating to cytokines and eicosanoids have been described, the immunomodulatory effects of miRNAs identified in NSC-CM are largely unexplored. miRNAs, such as miR-124 and let-7, are readily detected in NSC-CM and are thought to impart their non-cell-autonomous effects based on their immunomodulatory properties in other cell types (Ponomarev et al., 2011; Lv et al., 2018). However, few studies have directly explored the immunomodulatory effects of NSC-secreted miRNAs, which are predominately identified in EVs (Morton et al., 2018; Bian et al., 2020). Overcoming the technical barriers to studying miRNAs, and the EVs in which they are found will lead to a greater understanding of their immunomodulatory mechanisms.

In conclusion, studies identifying the exact factors within the NSC *secretome* that enable them to modulate the immune system are limited. Currently, only three factors (PGE2, TGF-β1, and TGF-β2) have been definitively identified. However, given the vast array of factors secreted by NSCs and the many contexts in which they can be applied, we have only yet scratched the surface of the NSC *secretome*. Promising candidates include other cytokines and bioactive lipids, additional miRNAs, as well as extracellular metabolites (Peruzzotti-Jametti et al., 2018).

A more thorough characterization of the NSC *secretome* is critical to better understand and, ultimately, harness the therapeutic potential of NSCs. Future studies aiming at uncovering immunomodulatory gene products would benefit from the inclusion of whole-genome microarrays and cytokine neutralization, an approach that has led to the identification of many neuroprotective factors found within the NSC *secretome* (Lee et al., 2017). Furthermore, parallel research utilizing the mesenchymal stem cell (MSC) *secretome* has confirmed the immunomodulatory properties of PGE2 and TGF-β (Salgado et al., 2015).

Thus, complimentary *secretome* findings from other stem cell sources will provide a powerful reference for the identification of similar factors within the NSC *secretome*. Finally, the advent of techniques such as SILAC will help identify NSC-secreted proteins (Prokhorova et al., 2009) and/or lipids (Stuani et al., 2018) in co-culture.

### NSC-EVs and Immune Responses

The release of biological signals within phospholipid-bound membranes could have originated as a method of eliminating damaged cellular components, and subsequently, may have provided a warning to local and distant cells acting as a primitive immune system (van Niel et al., 2018; Margolis and Sadovsky, 2019; Correa et al., 2020). EVs are now known to be critical mediators of intercellular communication and even act on the immune system and immune cells (Robbins and Morelli, 2014; Isola and Chen, 2017). Their cargo consists of antigens, cytokines, membrane proteins, microRNAs, and long/short noncoding RNAs. The make-up of the cargo is largely dependent on the cell of origin and can then be transferred to recipient cells *via* binding and internalization. Merging of the EV cytosol and the cell typically occurs through membrane fusion and endocytosis or by uptake through various pathways that involve the expression of specific proteins, including, but not limited to, tetraspanins, integrins, and proteoglycans (Mulcahy et al., 2014).

In the CNS, EVs are involved in cross-talk between multiple cell types, including neurons, astrocytes, microglia, oligodendrocytes, and infiltrating macrophages, where they normally participate in maintaining homeostasis by acting as signaling conduits, however they have also been identified as mediators of disease (Pegtel et al., 2014). Based on studies using NSC grafts in models of neurodegenerative conditions as well as *in vitro* culture work, the release of NSC-EVs not only dampens ongoing immune responses but activates regenerative programs (Camussi et al., 2013). The exact mechanism mediating the exchange of information between the secreted NSC-EVs and the eventual reaction of the recipient cell is still unknown and remains to be characterized. However, it is a rapidly developing field with multiple studies highlighting the impact of NSC-EVs on immune system function.

Microglia are influenced by endogenous NSC-EVs during neonatal development. EVs released by NSCs within the developing rodent subventricular zone (SVZ) contains an array of miRNAs that are found to regulate microglial physiology and morphology (Morton et al., 2018). Microglia uptake of NSC-EVs led to morphological changes and an altered transcriptional state represented by increased expression of genes related to inflammatory processes and the secretion of cytokines. Next-generation sequencing identified several members of the let-7 family, a miRNA precursor, to be highly enriched in NSC-EVs and responsible for phenotypic changes in microglia. Interestingly, the interaction of NSC-EVs and microglia generated a feedback loop, wherein the EV-treated microglia inhibited NSC proliferation *in vitro* by upregulated let-7-mediated cytokine release (Morton et al., 2018). This study suggests that NSC-EVs influence microglia regulate NSC proliferation during development through the modulation of inflammation-related genes.

Not only do NSC-EVs play a role in development but further research has identified the role of hypothalamic (ht) NSC-EVs in controlling the process of aging (Zhang et al., 2017). htNSC-EVs carry specific miRNAs that had been previously identified to substantially decrease in the cerebrospinal fluid during aging and linked to a decrease in overall physiological homeostasis. Transplantation of young htNSCs into aged mice recovered the optimal concentration of htNSC-EV-derived miRNAs and led to lifespan extension (Zhang et al., 2017). Therefore, the endocrine function of the hypothalamus involves the secretion of htNSC-EVs containing miRNAs which have potently modulate systemic aging. The exact function of the miRNAs is unknown, as well as the target cells of the htNSC-EVs, but one may speculate that the EVs target the immune system. Crosstalk between the hypothalamic neuroendocrine and immune system plays an important role in the regulation of homeostasis, however, an observed increase in inflammatory proteins from immune cells during aging further enhances physiological aging (Fulop et al., 2017). htNSC-EVs may target circulating inflammatory macrophages and T cells and dampen their reactivity, which has been observed in models of neuroinflammation.

In models of neuroinflammation, exogenous (injected) NSC-EVs are typically anti-inflammatory. EVs from NSCs derived from a human pluripotent stem cell line was found to improve a middle cerebral artery occlusion (MCAO) model of stroke in both mice and pigs when injected intravenously during the acute phase (Bacigaluppi et al., 2009, 2016; Bernstock et al., 2017, 2019; Webb et al., 2018a,b). NSC-EVs promoted a phenotypic switch in blood-borne macrophages from pro-inflammatory towards anti-inflammatory during the post-stroke phase. NSC-EVs also led to increased numbers of circulating regulatory T cells (T_reg_) which resulted in the downregulation of pro-inflammatory effector Th17 cells (Webb et al., 2018b). Overall, these two studies found that injected NSC-EVs provided neuroprotection in this model of stroke as evidenced by decreased lesion volume and altered inflammatory responses. One hypothesis is that NSC-EVs carry anti-inflammatory cytokines, such as IL-4 and IL-10, capable of promoting a phenotypic switch (Vogel et al., 2018). Another is that EV bound TGF-β and CD73 could inhibit T cell proliferation and activation (Anel et al., 2019). However, a direct main mechanism of action of NSC-EVs has not been yet identified (Hermann et al., 2014).

Within trauma-induced neuroinflammation, spinal cord injury (SCI) recovery is improved after rodent NSC-EV injection (Rong et al., 2019). NSC-EVs administered *via* the tail vein following SCI led to a significant reduction in the injured area along with a reduction in neuronal apoptosis, microglial activation, and neuroinflammation, which was attributed to the lower expression of pro-inflammatory cytokines TNF-α, interleukin 1-β (IL-1β), interleukin 6 (IL-6) and a decrease in activated microglia (Rong et al., 2019). Additionally, NSC-EVs prevented neuronal apoptosis through the beneficial promotion of neuronal autophagy when studied in a glutamate-induced neurotoxicity model *in vitro* (Tang et al., 2014; Rong et al., 2019). These beneficial effects were ablated following administration of an autophagy inhibitor, which suggests that NSC-EVs promote regeneration and a return to homeostasis through enhanced autophagic clearance of damaged cells *via* direct signaling onto neurons (Baixauli et al., 2014; Rong et al., 2019). Additional research is warranted to fully understand the direct effect and mechanisms of NSC-EVs on neurons. Further work in a rat model of SCI identified NOD-like receptor protein-3 (NLRP3), a key factor of inflammasome formation in the CNS, as a target of intrathecally injected rodent NSC-EVs (Mohammed et al., 2020). Inflammasomes are multiprotein intracellular complexes that become activated upon injury, stress, or infection and trigger pro-inflammatory cytokines to engage the innate immune response (Schroder and Tschopp, 2010). NSC-EV treatment of rats with experimental SCI suppressed the formation of the NLRP3 inflammasome complex, which led to reduced inflammation and enhanced recovery of motor function (Mohammed et al., 2020). Whether the enhanced motor function resulted from NSC-EVs acting directly or indirectly on motor neurons was not established. The cargo and defined mechanisms by which NSC-EVs promote this reduction in inflammation towards improved recovery in rodent models of SCI remains elusive.

Further work investigating the effect of NSC-EVs discovered their role in ameliorating inflammation in the transgenic amyloid precursor protein (APP)/presenilin 1 (PS1) mouse model of Alzheimer’s disease (AD; Li et al., 2020). Rodent NSC-EVs injected into the lateral ventricles of 9-month-old APP/PS1 transgenic mice gave rise to a significant improvement in overall cognitive behavior and mitochondrial function in the cortex that was accompanied by decreases in microglial activation. Most of these changes were attributed to an increase of the nicotinamide adenosine dinucleotide (NAD)-dependent deacetylase sirtuin 1 (SIRT1), a major regulator of metabolism, within the cortex following NSC-EV treatment (Li et al., 2020). Here SIRT1 is thought to contribute to the anti-inflammatory behavior of microglia by inhibiting the epigenetic regulation of pro-inflammatory cytokines, including IL-1β, and restoring normal mitochondrial metabolism (Cho et al., 2015; Tang, 2016; Peruzzotti-Jametti and Pluchino, 2018).

The common theme of NSC-EVs is their potent ability to promote context-dependent anti-inflammatory responses. However, the overall mechanisms are largely unknown, and of those with identified mechanisms, only a handful have profiled their cargo and target cells. One such study demonstrated that the treatment of rodent NSCs with pro-inflammatory cytokines stimulated the interferon-gamma (IFN-γ)-Stat1 signaling pathway that resulted in the export of EVs with cargo that mirrored the cellular contents (Cossetti et al., 2014). IFN-γ was found bound to the interferon-gamma receptor 1 (IFNGR1) on the surface of NSC-EVs, which then activated Stat1-dependent signaling on target cells *via* the intracellular transfer of IFN-γ. This work demonstrates how EVs signal to their target cells *via* membrane interactions (Cossetti et al., 2014). Subsequent work went on to show that NSC-EVs behave as independent metabolic units carrying L-asparaginase activity, suggesting that NSCs act as metabolic regulators of distant cells (Iraci et al., 2017). By altering the metabolic aspects of the microenvironment, NSC-EVs also may shape the phenotype of surrounding immune cells (Drago et al., 2016).

Within the retina, NSC-EVs can protect photoreceptor cells in a rat model of inherited retinal degeneration by microglial internalization (Bian et al., 2020). Here, subretinal injection of rodent NSC-EVs tagged with a CD63-red fluorescent protein (RFP) were specifically internalized by retinal microglia. Microglia with internalized NSC-EVs displayed a ramified, homeostatic morphology compared to the rounded, activated morphology of NSC-EV absent microglia. Analysis of the NSC-EVs using small RNA-sequencing identified a set of 17 miRNAs implicated in the targeting of TNF-α, IL-1β, and cyclooxygenase-2 (COX2), all known activators of microglia. *in vitro* modeling confirmed decreases in these pro-inflammatory factors upon microglial NSC-EV internalization. This work suggests that NSC-EVs can directly target the immune system and release miRNAs capable of suppressing activated microglia and restoring a neuroprotective environment (Bian et al., 2020).

Understanding the kinetics of EV release, their cargo loading, and functional effects on other cells is a highly complex task. NSCs release a variety of EV subtypes that are dependent on their current cell state, often in response to microenvironment stressors such as cytokines/chemokines. They have also been identified as homeostatic regulators that control developmental and aging mechanisms (Vogel et al., 2018). Further, many current studies have identified a beneficial role of NSC-EVs in abrogating inflammation but have failed to identify the mechanism by which this occurs. On the one hand, it could be attributed to their miRNA, RNA, and protein cargo that target and enter cells, in turn changing their phenotype. On the other hand, EV signaling receptor mechanisms on the EV membrane and the membrane of the target cell may also lead to downstream pathways inducing overall cellular changes.

Research towards understanding these important mechanisms is warranted to identify how NSC-EVs could be used to alter immune system responses in disease to enhance recovery.

### Approaches for Studying the NSC-Secretome in Regenerative Neuroimmunology

Determining how the NSC *secretome* alters immune cell function can be further parsed by using *in vitro*-based approaches. Over the past decade, we have seen advancements in the ease of culturing both rodent and human NSCs as well as the ability to perform genetic manipulation, which can provide proof-of-concept evidence of secreted factors. Proteomics on CM from NSCs can catalog what molecules and pathways the *secretome* may target, but further elucidating how the *secretome* can modulate immune cells requires additional investigation. For example, NSCs can be used to model neurodegenerative diseases, such as progressive multiple sclerosis (PMS). Proteomics on human PMS NSCs *in vitro* unveiled high expression of high mobility group box 1 (HMGB1), which can act as a pro-inflammatory alarmin by binding to toll-like receptors (TLRs) on microglia to perpetuate chronic inflammation (Nicaise et al., 2019). Unfortunately, this methodology only reveals cell-secreted factors in isolation from the inflammatory environment, which may change what the NSCs secrete in response when in contact with immune cells in the *in vivo* setting.

To further delve into mechanisms involving the crosstalk of immune cells and NSCs, co-culture systems have been used. Non-contact trans-well systems allow for secreted factors to pass through and communicate with immune cells to provide mechanistic evidence for this relationship. Using this trans-well system, co-cultures of LPS pre-treated microglia with rodent NSCs were found to modulate LPS-activated microglia by suppressing inflammation. Using targeted ELISAs of the CM for several immune factors identified the chemokine C-X-C Motif Chemokine Ligand 12 (CXCL12)/stromal cell-derived factor 1 (SDF1)-α as significantly decreased upon NSC co-culture (Gao et al., 2017). This suggests CXCL12 binds to its cognate receptor CXCR4 on NSCs to modulate microglial activation states, as RNA silencing of *Cxcr4* expression in NSCs ablates the anti-inflammatory effects on activated microglia in this system (Gao et al., 2017).

Overall, *in vitro* approaches provide a more mechanistic approach for studying interactions between the NSC *secretome* and immune cells. It allows researchers to directly study proteins, metabolites, and even EVs being secreted by NSCs within defined contexts. Biased, such as ELISAs, and unbiased, including proteomics, analyses can be used to profile secreted factors within the co-culture system. Downstream genetic deletion or receptor blocking can be performed to determine the mechanism of action. However, major shortcomings associated with *in vitro* work remain as it does not completely replicate the unique *in vivo* environment accompanying NSC transplants nor the dynamic interactions with other cell types, such as astrocytes, oligodendrocytes, neurons, and immune cells. Despite this, *in vitro* model systems provide valuable information regarding the interactions of the NSC *secretome* with immune cells.

Targeting and diminishing chronic neuroinflammation remains a key goal in many therapeutic approaches to neuroregeneration. NSCs, and the NSC *secretome*, have repeatedly demonstrated their anti-inflammatory effects across multiple models of neurodegenerative disorders and several mammalian species after transplantation *in vivo*. The most common animal models of neurodegeneration that have been targeted with NSCs are rodents but other prevalent *in vivo* models include pigs (Webb et al., 2018a) and non-human primates (Redmond et al., 2007; Rosenzweig et al., 2018). The immunomodulatory capacity of NSCs has been harnessed through a wide range of transplantation routes. More recently, however, treatment with NSC-CM only retains many of the therapeutic benefits of whole NSCs while bypassing many of the risk factors associated with transplantation, namely tumorigenicity, and immunogenicity (Mousavinejad et al., 2016). Here, we will discuss select studies that have exemplified the immunomodulatory effects of NSCs and NSC-CM in the context of animal models of neurodegeneration.

NSCs have been extensively trialed in the quest to regenerate axons after SCI. However, it has become increasingly clear that the indefinite persistence of cytotoxic inflammation is key to inhibiting regeneration after SCI injury. Thus, the promise of NSCs in SCI has shifted from one of cell replacement to that of microenvironment modulation.

Mouse fetal NSCs transplanted into the spinal cord of murine models of contusion SCI at 7 days post-injury were found to be viable and capable of migrating towards the lesion core (Cheng et al., 2016). In this setting, the anti-inflammatory effects of the NSC transplant were found to reduce infiltrating myeloid cells, possibly through a similar reduction in pro-inflammatory cytokine (*Tnf, Il1b, Il-6, and Il-12*) expression. Moving *in vitro*, co-culture of BMDMs, and NSCs before stimulation with IFN-γ significantly inhibited the expression of IFN-γ-responsive pro-inflammatory cytokines (*Tnf, Il1b, Il-6, Il-10, and iNos*) from BMDMs. Thus, they concluded that NSCs play an anti-inflammatory role in SCI by inhibiting the pro-inflammatory activation of macrophages, similar to what is described in mice with EAE (Peruzzotti-Jametti et al., 2018).

Given the clinical limitations in transplanting NSCs, such as the limited long-term viability of the NSC graft, the same group endeavored to investigate the anti-inflammatory effects of fetal murine NSC-CM in the context of SCI (Cheng et al., 2017). In this study, *in vitro* LPS-activated BMDMs were treated with NSC-CM which reduced the expression of pro-inflammatory cytokines (*Il-6, Il-12, and* induced nitric oxide synthase [*iNos*]) to near basal levels. Moving *in vivo*, NSC-CM administered intraperitoneally following experimental murine SCI led to the downregulation of *Il-1b, Il-6, and iNos* while upregulating *Il-12*. Further, the systemic application of NSC-CM not only improved functional outcomes and reduced the lesion volume but also decreased systemic inflammation through reduced iNOS production. This suggests the overall effect of NSC-CM is anti-inflammatory and pro-regenerative in this experimental injury setting and, importantly, suggests the anti-inflammatory effects of the NSC *secretome* are systemic.

Identifying a systemic benefit of the NSC *secretome* on immune system activation is an alluring premise. In rats with experimental sciatic nerve injury, which affects the peripheral nervous system, IV injection of CM from human embryo-derived NSCs provided promising results (Chen et al., 2020). Here, continual treatment with hNSC-CM in injured rats *in vivo* did not alter the acute, Schwann cell-driven inflammation, rather it abrogated the macrophage-driven inflammatory response in the chronic stage through a reduction in the expression of pro-inflammatory cytokines *Tnf*, *Il6*, and *Il1b* and the accumulation of CD68^+^ macrophages. This finding supports other lines of investigations into the effects of the NSC *secretome* on macrophages in the CNS (Huang et al., 2014; Cheng et al., 2017; Peruzzotti-Jametti et al., 2018). To determine if the anti-inflammatory effects of hNSC-CM were specific to macrophages, rat peritoneal macrophages treated with hNSC-CM had decreased expression of both pro-inflammatory cytokines (*Tnf, Il6, and Il1b*) and the pro-inflammatory enzyme *iNos*, in LPS activated macrophages. Key to this effect was the activation of the SIRT-1 signaling pathway as blocking the activation of SIRT-1 in LPS treated macrophages allowed for the activation of the downstream transcription factors NF-kB and HIF-1α and induction of pro-inflammatory genes *Tnf, Il6, and Il1b* and *N*os2. Similar findings have been demonstrated in the BV-2 microglial cell line (Ye et al., 2013). Although this study failed to discover the mechanism through which the hNSC *secretome* activates the Sirt-1 pathway, a plausible candidate is TGF-β1, which demonstrates anti-inflammatory effects (Chen et al., 2014), activates Sirt-1 (Cha et al., 2016), and is present in the hNSC *secretome*.

Ischemic stroke is one of the three most prevalent causes of death and disability that disproportionally targets the rapidly increasing aged population (Zhang et al., 2019). Similar to other neurodegenerative diseases, the hope of NSC transplants as a therapeutic option in ischemic stroke is twofold: (i) to repopulate the lesion site through cell differentiation and neurogenesis, and tissue repair; and (ii) to modulate the pro-inflammatory microenvironment.

A major impediment to the applicability of NSC in the context of ischemic stroke is ensuring that the transplants correctly migrate to the lesion to impart their beneficial regenerative and immunomodulatory functions. In murine models of MCAO to induce focal ischemia followed by reperfusion (MCAO/R), the injection of fetal hNSCs into the ipsilesional hippocampus, a region with endogenous migration cues, promoted the rapid migration of the grafts to the lesion epicenter (Huang et al., 2014). To investigate the immunomodulatory effects of the hNSCs, they were delivered at the height of the pro-inflammatory cytokine response (24 h post-injury). At 48-h post-injury, hNSCs were observed migrating to the infarct which correlated with reduced infarct volumes and improved behavioral outcomes. They concluded that this rapid effect was caused by the anti-inflammatory properties of the hNSCs, as they observed a reduction in microglial activation and downregulation of the transcripts of pro-inflammatory cytokines (*Tnf*, *Il6*, *Il1b*, *Ccl2*, *Ccl3*) from brain homogenate.

Another challenge in the use of NSCs as a treatment for ischemic stroke is the invasive nature of the delivery route, namely intracerebral injection. An alternative route for NSC delivery into the lesion site is through epidural injection, which was demonstrated to be effective 1 week after permanent MCAO (Lee et al., 2017). Despite poor migration and viability of the human iPSC derived NSC grafts, improved functional outcomes, reduced ED1^+^ myeloid progenitor cells, and astrogliosis, increased angiogenesis, and reduced lesion volume at 21 days post-injury was observed. Thus, the observed improvements were postulated to be due to the paracrine effects of the grafted NSCs leading them to conduct *in vitro* whole-genome microarrays and cytokine neutralization experiments to identify the NSC secreted factors. Here, the focus of their subsequent experiments focused on the neuroprotective ability of five factors (bone morphogenetic protein 7, chemokine ligand 14, fibroblast growth factor 8, fibroblast growth factor 9, and insulin-like growth-factor-binding protein 2), but further investigation of their NSC *secretome* data would likely have identified candidates responsible for the anti-inflammatory mechanisms and reduction in myeloid cell recruitment.

The numerous issues associated with cell therapy, including invasive intracerebral injection and ensuring rapid NSC migration to the lesion core, comprise significant hurdles for NSC transplants as a therapeutic approach for ischemic stroke, despite the beneficial clinical effects in experimental animal models. To overcome these limitations, CM from fetal rat NSCs was delivered *via* vein tail injections at three consecutive time points (3, 24, 48 h post-injury) in rat models of MCAO/R (Yang et al., 2018). Similar to findings in studies using NSC transplants, the systemic application of NSC-CM reduced infarct volume and improved behavioral outcomes and mitochondrial ultrastructure, which was suggested to be partially due to a reduction in inflammation, although they did not investigate this line of thinking any further.

The leading cause of non-traumatic disability in young adults is MS, an inflammatory demyelinating disease of the CNS (Wallin et al., 2019). As in the previously discussed neurodegenerative pathologies, the therapeutic potential of NSCs in MS arises from: (i) their neuroprotective and regenerative properties; and (ii) their endogenous anti-inflammatory properties.

Given the persistent inflammation present in progressive MS, and the clear immunomodulatory effects of NSCs, it is not wholly unsurprising that NSCs have been extensively studied within the context of this disease. In 2003, we demonstrated that intracerebroventricular and intravenous delivery of adult rodent NSCs ameliorated the behavior and pathophysiological deficits observed in murine EAE models of MS (Pluchino et al., 2003). Over a decade later, it was shown that this effect holds for murine iPSC-derived NSCs and that intraventricular transplants also decrease T-cell infiltration (Zhang et al., 2016). However, other studies did not observe this decrease in T-cell infiltration in the EAE model, possibly due to differences in the time point of transplantation, number and concentration of transplanted NSCs, and type of EAE performed (Peruzzotti-Jametti et al., 2018).

Unlike in other disease models, the study of NSC transplantation in animal models of MS has delved into their immunomodulatory effects. For example, the studies discussed in the previous section were able to pinpoint the immunomodulatory factors that played a role in either shifting the inflammatory profiles of macrophages (De Feo et al., 2017; Peruzzotti-Jametti et al., 2018) or T-cells (Chen et al., 2014; Plaisted et al., 2016) and each of these studies was performed in a model of MS. However, a potential gap in the MS-NSC immunomodulatory literature is the evaluation of NSC-CM in animal models of MS. Given the immunomodulatory effects observed in other disease models, further investigation into this approach, possibly in combination with NSC transplants, might represent an even further enhanced therapeutic potential for NSCs in the context of MS.

Beyond the neurodegenerative diseases described, NSCs and NSC-CM have revealed similar anti-inflammatory effects and improved functional outcomes in several other CNS pathologies, including Parkinson’s disease (Redmond et al., 2007; Mendes-Pinheiro et al., 2018), amyotrophic lateral sclerosis (Zalfa et al., 2019), and AD (Lee et al., 2015; McGinley et al., 2018). Thus, the application of NSCs or the NSC *secretome*, harnessed *via* NSC-CM, has shown promising results across a diverse array of *in vivo* models of neurodegeneration. Common themes include cross-talk between NSCs and endogenous immune cells, which generally results in either a decrease in inflammatory immune cell infiltration or a shift in their inflammatory profiles and the ability of NSC-CM to elicit immunomodulatory effects. However, many of these effects have yet to be carefully compared within the same study. Unsurprisingly, the anti-inflammatory effects observed from the application NSCs/NSC-CM involves the downregulation of the targets of the NF-kB pathway, suggesting the possibility of NF-kB antagonist(s) within the NSC *secretome*. Overall, the immunological effects of NSCs in neural regeneration is overwhelmingly anti-inflammatory. Thus, given that persistent neuroinflammation contributes to the chronic pathology of multiple CNS diseases, harnessing the anti-inflammatory properties of the NSC *secretome* represents a promising approach for therapeutic intervention.

## Implications for Clinical Work

The anti-inflammatory effects of stem cells (including NSCs) may be of value in conditions in which the immune system becomes hyperreactive as a consequence of initial activation. In the event of tissue damage, whether caused by injury or infection, the immune system produces and releases multiple inflammatory cytokines (such as TNF-α, IL-1β, IL-8, and IL-6) to clear the site of inflammation and recruit additional immune responses. However, in some cases, this cytokine release can become uncontrolled and lead to prolonged intensified inflammation-causing leakage from capillaries, tissue oedema, and shock (Bird, 2018). This is known as *cytokine storm* and it has been previously described as a consequence of both viral and bacterial infections (e.g., influenza A and Francisella tularensis; D’Elia et al., 2013), and more recently it has become a pathophysiological aspect of incredible importance in the current coronavirus disease 19 (COVID-19) pandemic (Wiersinga et al., 2020).

Preliminary clinical data indicate that severe acute respiratory syndrome coronavirus 2 (SARS-CoV-2) infection is associated with an intense cytokine storm in some patients. A multiplex screen for 48 cytokines of COVID-19 patients has indeed shown a marked increase of pro-inflammatory cytokines in patients with clinically moderate and severe COVID-19, compared with healthy controls (Yang et al., 2020). Continuously high levels of these cytokines (especially CXCL10, CCL7, and IL-1 receptor antagonist) are associated with increased viral load, loss of lung function, lung injury, and a fatal outcome.

Several therapeutic strategies have been trialed to bring the inflammatory response back under control in COVID-19 patients. The UK Randomised Evaluation of COVID-19 therapy (RECOVERY) trial, which is comparing a range of possible treatments with usual care in patients hospitalized with COVID-19, has shown clear benefits of dexamethasone treatment (Horby et al., 2020). A preliminary report found that intravenous dexamethasone reduces deaths by a third in patients receiving invasive mechanical ventilation (29.3% vs. 41.4%%, rate ratio, 0.64; 95% CI, 0.51–0.81), and by one fifth in patients receiving oxygen without invasive mechanical ventilation 23.3% vs. 26.2%; rate ratio, 0.82; 95% CI, 0.72–0.94), but not among those who are receiving no respiratory support at randomization (17.8% vs. 14.0%; rate ratio, 1.19; 95% CI, 0.91–1.55). The use of tocilizumab, a marketed IL-6 blocking antibody, has also been shown to be beneficial in an Italian retrospective, observational cohort study (Guaraldi et al., 2020). Tocilizumab treatment is associated with a reduced risk of invasive mechanical ventilation or death (adjusted hazard ratio 0.61, 95% CI 0.40–0.92; *p* = 0.020). Of note, 24 (13%) of 179 patients treated with tocilizumab were diagnosed with new infections, vs. 14 (4%) of 365 patients treated with standard of care alone (*p* < 0.0001).

Promising results were also obtained in the Netherlands, where researchers of the COVID High-intensity Immunosuppression in cytokine storm syndrome (CHIC) study showed that a strategy involving a course of high-dose methylprednisolone, followed by tocilizumab if needed (vs. a strategy with supportive care only) in patients with COVID-19 led to a clinically relevant improvement of respiratory status, a reduction of the hospital mortality, and the need for mechanical ventilation (Ramiro et al., 2020).

These clinical trials suggest that the *cytokine storm* is indeed a treatable complication of COVID-19 and at this point, high-dose glucocorticoids and immunosuppressive treatments may be the best option for patients.

**Figure 2:**
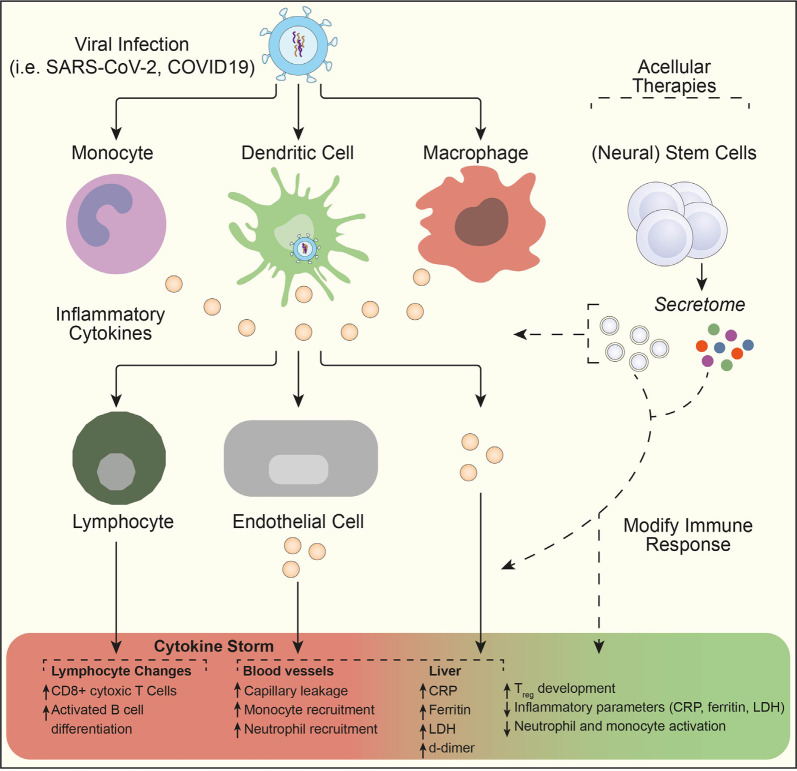
NSC *secretome* as a potential acellular therapeutic in the treatment of cytokine storm. Viral infection results in monocytes, dendritic cells, and macrophage activation. The release of pro-inflammatory cytokines can then initiate an amplification cascade that results in cytotoxic T lymphocyte differentiation, monocyte and neutrophil recruitment, and increased circulating inflammatory parameters. Subsequent enhanced systemic cytokine production contributes to the pathophysiology of severe viral infections (such as SARS-CoV-2 and COVID-19) through a cytokine storm. Here, acellular therapeutic intervention using the stem cell *secretome* might lead to modification of the immune response and a rapid dampening of pro-inflammatory activity through increased T_reg_ development. Figure adapted from Moore and June (Moore and June, 2020). Abbreviations: CRP, C-reactive protein; LDH, lactate dehydrogenase; T_reg_, regulatory T cell.

Stem cell therapies and, more recently, their secreted (EVs) are emerging as promising treatments, which could also attenuate inflammation and regenerate the lung damage caused by COVID-19 (Chrzanowski et al., 2020). There are currently >15 clinical trials evaluating the therapeutic potential of mesenchymal stem cells (MSCs) for the treatment of COVID-19, the majority of which rely on intravenous administration. While the outcomes from most of these trials have yet to be reported, a proof of concept study with adipose-tissue (AT) MSCs (AT-MSC) suggests that this treatment is safe and potentially useful in severe cases of COVID-19 requiring mechanical ventilation (Sánchez-Guijo et al., 2020). With a median follow-up of 16 days after the first dose, clinical improvement was observed in 70% of patients receiving allogenic AT-MSC, and treatment was followed by a decrease in inflammatory parameters (C-reactive protein, IL-6, ferritin, LDH, and d-dimer), particularly in those patients with clinical improvement. The *secretome* of stem cells can be also an effective treatment option for COVID-19 cytokine storm. One clinical trial (NCT04276987) is currently testing MSC-derived exosomes *via* inhalation route in patients with severe pneumonia arising from COVID-19 infection. The main advantages of this acellular approach are the lower risk of mutagenicity and oncogenicity (as there is no cell graft) and the stability of the medicinal product, which can be easily transported and administered (Figure 2).

Besides its peripheral effects, the COVID-19 cytokine storm may lead to the direct involvement of the nervous system causing: (i) encephalopathies; (ii) inflammatory CNS syndromes; (iii) ischemeic strokes; (iv) peripheral neurological disorders; and (v) miscellaneous CNS disorders (Paterson et al., 2020). COVID-19 infection is indeed associated with a wide spectrum of neurological syndromes affecting the whole neuroaxis, leading to a strikingly high incidence of acute disseminated encephalomyelitis, which is not related to the severity of the respiratory COVID-19 disease. While some of these cases respond to immunotherapies (corticosteroids or intravenous immunoglobulin), their prognosis is still poor. Since, the underlying mechanisms of this encephalopathy may result from the combined effects of sepsis, hypoxia, and immune hyperstimulation due to the cytokine storm (Mehta et al., 2020), the use of stem cells and their *secretome* could prove to be extremely useful in these cases.

In this sense, treatment with NSC-EVs could provide an additional benefit, given its previously described immunomodulatory effects and its CNS tissue specificity. Further trials with allogeneic NSC *secretome* will be needed to explore this possibility and its application in these clinical settings.

## Conclusion

There are still several questions that need to be addressed when it comes to harnessing the NSC *secretome* for Regenerative Neuroimmunology. First and foremost, most studies lack mechanistic identification of the effect of secreted factors, along with profiling EV contents. Future work is needed to delve into and profile NSC factors that could be used in promoting regeneration, and how exactly these factors are altering the inflammatory environment. This involves study in performing targeted proteomics and sequencing on NSC-derived factors, in addition to the isolation and analysis of NSC-EVs, in the context of various inflammatory conditions. Generating a larger panel of NSC secreted factors will help in future large-scale high-throughput assays which can assess the utility of these factors on immune cells. Harnessing these factors will allow for a more directed therapeutic, rather than relying on the transplantation of NSCs, which faces concerns of tumor formation and immune rejection. Further, the NSC *secretome* may play larger roles in non-CNS specific conditions, such as the targeting of cytokine storms, known to occur with COVID-19. Overall, the continuation in basic research towards further understanding the *secretome* of NSCs is warranted in the search for new regenerative strategies.

## Author Contributions

CW designed review outline, wrote manuscript, and designed figures. AN, RH, VP, and LP-J contributed sections of manuscript. AN, LP-J, and SP critically reviewed manuscript. All authors contributed to the article and approved the submitted version.

## Conflict of Interest

SP is co-founder and CSO at CITC Ltd. and iSTEM Therapeutics, and co-founder and Non-executive Director at asitia Therapeutics. LP-J is Director of Strategy and Innovation at CITC Limited. AN is an advisor for iSTEM Therapeutics. The remaining authors declare that the research was conducted in the absence of any commercial or financial relationships that could be construed as a potential conflict of interest.
